# A Review of Transition Metal Dichalcogenides-Based Biosensors

**DOI:** 10.3389/fbioe.2022.941135

**Published:** 2022-06-13

**Authors:** Hongyu Sun, Dujuan Li, Xiaojie Yue, Rui Hong, Weihuang Yang, Chaoran Liu, Hong Xu, Jun Lu, Linxi Dong, Gaofeng Wang, Dongyang Li

**Affiliations:** ^1^ Ministry of Education Engineering Research Center of Smart Microsensors and Microsystems, School of Electronic Information, Hangzhou Dianzi University, Hangzhou, China; ^2^ School of Automation, Hangzhou Dianzi University, Hangzhou, China; ^3^ The Children’s Hospital of Zhejiang University School of Medicine, Hangzhou, China; ^4^ College of Life Sciences and Oceanography, Shenzhen University, Shenzhen, China; ^5^ School of Science, Faculty of Health and Environmental Sciences, Auckland University of Technology, Auckland, New Zealand; ^6^ Laboratory of Agricultural Information Intelligent Sensing, College of Biosystems Engineering and Food Science, Zhejiang University, Hangzhou, China

**Keywords:** transition metal dichalcogenides (TMDCs), biosensor, non-covalent, covalent interaction, modification methods

## Abstract

Transition metal dichalcogenides (TMDCs) are widely used in biosensing applications due to their excellent physical and chemical properties. Due to the properties of biomaterial targets, the biggest challenge that biosensors face now is how to improve the sensitivity and stability. A lot of materials had been used to enhance the target signal. Among them, TMDCs show excellent performance in enhancing biosensing signals because of their metallic and semi-conducting electrical capabilities, tunable band gap, large specific surface area and so on. Here, we review different functionalization methods and research progress of TMDCs-based biosensors. The modification methods of TMDCs for biosensor fabrication mainly include two strategies: non-covalent and covalent interaction. The article summarizes the advantages and disadvantages of different modification strategies and their effects on biosensing performance. The authors present the challenges and issues that TMDCs need to be addressed in biosensor applications. Finally, the review expresses the positive application prospects of TMDCs-based biosensors in the future.

## 1 Introduction

**FIGURE 1:**
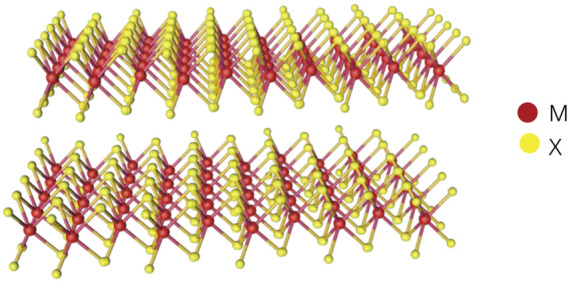
The three-dimensional structure of TMDCs where M is transition metal element and X is chalcogen element.

Nanomaterials can be divided into four categories according to their dimensionality, including zero-dimensional materials, one-dimensional materials, two-dimensional materials (2DMs) and three-dimensional materials (Liu et al., 2016; Chen et al., 2021). In biomedicine field, 2DMs have broad application prospects (Kurapati et al., 2016). Transition metal dichalcogenides (TMDCs), as an emerging 2DMs, have attracted great interest due to its excellent physical and chemical properties. As shown in the chemical structure in Figure 1, TMDCs are a sandwich structure with chalcogen atoms separated by a plane of metal atoms in two hexagonal planes. The atoms in these layers are firmly bonded together by covalent bonds, while each thin layer is connected by relatively weak van der Waals forces (Wang et al., 2017; Wang et al., 2020a; Rahman et al., 2021).

The atomic layers of TMDCs can be easily separated from each other to form atomic-level sheets because they are held by weakly bonded van der Waals forces (Monga et al., 2021). Several methods have been used to prepare single- or few-layer 2D-TMDCs nanosheets, which can broadly be divided into top-down and bottom-up approaches. Top-down methods (Wang et al., 2017) aim at transforming bulk crystals and layered compounds into single- and few-layer 2D-TMDCs including mechanical exfoliation and liquid exfoliation. The mechanical exfoliation refers to peel off single layer or a few layers of flakes from bulk TMDCs by scotch tape. This method is cheap, simple to operate, and high in quality (Zhao et al., 2020). However, the TMDCs flakes fabricated by this method are limited to scientific research applications due to the uncontrolled shape, size, and thickness (Zhu et al., 2019). The liquid exfoliation methods refer to sonication in aqueous solution or addition of surfactant to exfoliate TMDCs (Rohaizad et al., 2021). This method is suitable for large-scale production (Vega-Mayoral et al., 2016), but rarely produces monolayer of TMDCs nanosheets (Zhou et al., 2020). Bottom-up approaches refer to the growth of layered nanomaterials under special conditions with atoms or molecules as precursors (Zhao et al., 2020). As a typical example, chemical vapor deposition (CVD) is a more promising method for preparing large-scale, continuous, and uniform single- or few-layer TMDCs films (Xiong et al., 2020; Liu et al., 2020a). However, the performance of TMDCs prepared by this way still need to be improved. For example, amorphous precursors will be used in the exfoliation process. This will result in the obtained TMDCs films being mostly polycrystalline with small grain size, and the grain boundaries in these polycrystalline films will greatly reduce the electrical properties of TMDCs (Li et al., 2021b; Kim et al., 2021a). Therefore, it is still a challenge to achieve the large-scale preparation of high-performance, low-defect monolayer and few-layer TMDCs nanosheets (Hu et al., 2019).

As the film thickness decreases, TMDCs can exhibit different properties (Ambrosi et al., 2015). 2D-TMDCs exhibite the transition of an indirect bandgap to a direct bandgap when bulk materials are scaled down to monolayers, accompanied by unique electrical and optical properties (Choi et al., 2017). A material with direct bandgap have better light utilization and can produce unique optical and electrical properties, making them ideal for a variety of optoelectronic devices (Monga et al., 2021). It had been demonstrated that a single layer of MoS_2_ can provide an on/off ratio of >10^8^ when applied to field effect transistor configurations, a value that is very favorable for establishing biosensors (Kalantar-zadeh and Ou, 2015). With the excellent electrical properties of MoS_2_, a MoS_2_ field-effect transistor sensor array was constructed to detect two bladder cancer markers, nuclear matrix protein 22 (NMP22) and cytokeratin 8 (CK8) simultaneously, with ultra-low detection limits of 0.027 and 0.019 aM achieved, respectively (Yang et al., 2020); A biosensor based on bilayer MoS_2_ back-gate field-effect transistor can detect glucose solution with high sensitivity, with a lower detection limit of 300 nM achieved (Shan et al., 2018).

The high flexibility of ultrathin TMDCs nanomaterials enabled them to be easily deposited onto flexible substrates. At the same time, the mechanical strength of TMDCs enabled them to be adapted well to the human body. These characteristics made it promising for wearable and implantable biosensor devices (Sarkar et al., 2014; Choi et al., 2019). An e-skin compatible humidity sensor was synthesized by metal sulfurization based on a large-area polycrystalline few layers WS_2_ film. The sensor can withstand tensile strain loads of up to 40% and still show a repeatable humidity response under tensile loading states (Guo et al., 2017). A prebent MoS_2_ structures were prepared on flexible substrates using a sacrificial structure-assisted nanofabrication method. This method was able to precisely control the bending curvature and position of the prebent MoS_2_ structures. The sensor was able to detect interleukin-1β (IL-1β) successfully as low as 10 fM (Ryu et al., 2017).

As discussed above, TMDCs have been successfully applied in biosensors due to their unique properties. Biosensor is a device that is sensitive to biological substances and can convert their concentrations into electrical signals for detection (Hong et al., 2022). It mainly consists of two parts: molecular recognition element and transducer (Aydin et al., 2018). Biosensors are widely used in the research of life and health due to their sensitivity and specificity. However, the biggest challenge for biosensors is still to further improve the sensitivity to meet the needs of ultra-low target concentration in practical applications (Mani et al., 2021). A lot of materials such as silicon nanowires (Li et al., 2021a), graphene oxide (Verma and Singh, 2019), TMDCs (Li et al., 2019) have been used to enhance the target signal. Among them, due to their good metallic and semi-conducting electrical capabilities (Kim et al., 2020a), tunable bandgap (Zhang et al., 2019a) and the large specific surface area, TMDCs show excellent performance in enhancing biosensing signals.

The functionalization of TMDCs is a critical step for the fabrication of TMDCs-based biosensors. Although the lack of dangling bonds on the surface of TMDCs brings difficulties to the fabrication of TMDCs-based biosensors, other properties of TMDCs play a key role in its surface modification. For example, the van der Waals forces of their surface, or some defects (Li et al., 2022a) of their own, such as sulfur vacancies (Chou et al., 2013); or the addition of surfactants to aqueous solution (Backes et al., 2016). What’s more, we could cover the surface of TMDCs with a layer of intermediates, such as hafnium oxide (HfO_2_) (Masurkar et al., 2021) or alumina (Al_2_O_3_) (Park et al., 2017), which can be easily modified with silanes. Therefore, it can provide abundant aldehyde groups for sensor fabrication. All the above properties can be used for the TMDCs surface modification to realize the fabrication of TMDCs-based biosensors.

**FIGURE 2:**
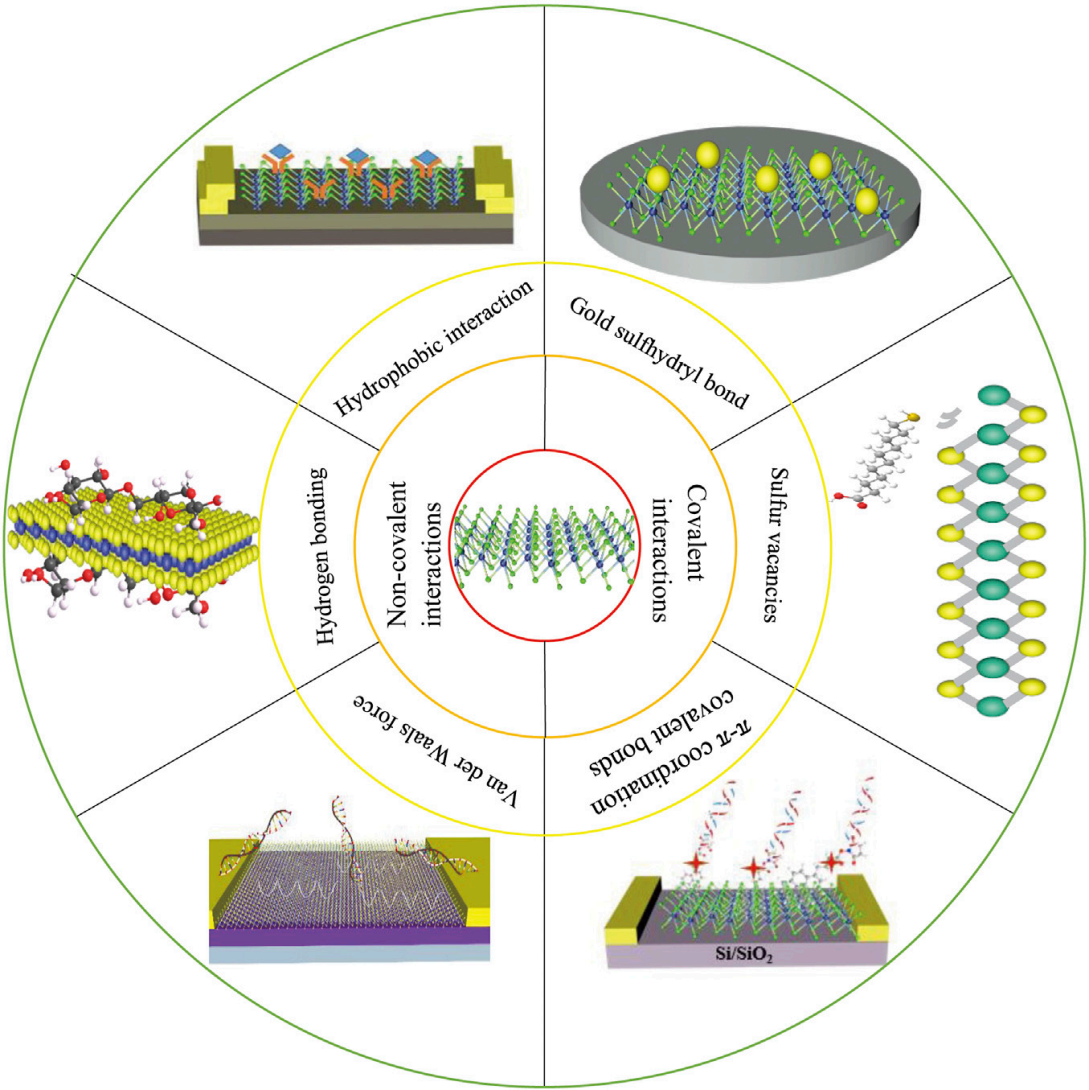
Summary of the surface modification methods and biosensor applications of TMDCs.

This review summarizes the surface modification methods of TMDCs represented by MoS_2_ and WS_2_, and the research progress of TMDCs-based biosensors (Figure 2). The advantages and disadvantages of biosensors based on different TMDCs modification methods are discussed. Authors also present the challenges and issues that need to be addressed. Finally, the review expresses the positive applications prospects of TMDCs-based biosensors in the future.

## 2 Biosensors Based on Non-Covalent Interactions on the Surface of Transition Metal Dichalcogenides

The special sandwich-like structure of TMDCs allows effective adsorption of various molecules through non-covalent interactions. Among the non-covalent interactions, hydrophobic interaction, van der Waals force, and hydrogen bonding are well studied (Kumar et al., 2021). For example, TMDCs can directly adsorb antibodies *via* hydrophobic interaction (Das et al., 2020) and single-stranded DNA molecules (Kumar et al., 2022), gas molecules (Bharathi et al., 2022), and silane coupling agent through van der Waals force. TMDCs can also be linked with surfactants (Jung et al., 2018; Parra-Alfambra et al., 2018) such as dextran through hydrogen bonding, enabling simultaneous exfoliation and surface functionalization of multilayer nanosheets.

### 2.1 Hydrophobic Interaction

Hydrophobic interactions are mediated by water (Garde, 2015; Jia et al., 2022). The mutual repulsion of the hydrophobic segments and water led to the proximity of the hydrophobic parts of the system to each other (Mozo-Villarias et al., 2021). All types of proteins such as antibodies inevitably contain a large number of hydrophobic segments (Zhou et al., 2022). Therefore, the presence of “hydrophobic interactions” in the “aqueous”systems makes it easier for antibodies containing hydrophobic segments to adhere to the surface of hydrophobic substrates. Therefore, the hydrophobic interface is more conducive to direct physical adsorption of antibodies. This is important for the construction of biosensors, because the additional complexity involved in chemical treatment can be avoided (Dzupponova and Zoldak, 2021).

Hydrophobicity is mainly characterized by the surface contact angle of the substance. The MoS_2_ surface has a relatively high contact angle of ∼75.77° with good hydrophobicity (Gaur et al., 2014), allowing a higher affinity for biomolecules such as antibodies.

**FIGURE 3:**
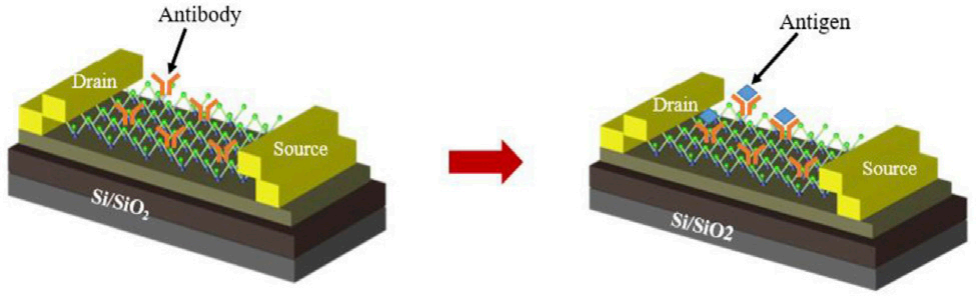
Schematic diagram of MoS_2_ biosensor for PSA detection (Lee et al., 2014).

Based on the hydrophobic interaction, Lee et al. (2014) studied a MoS_2_-FET biosensor to detect prostate specific antigen (PSA) in a highly sensitive and label-free manner. In the construction of the sensor, MoS_2_ nanosheets prepared by mechanical exfoliation were transferred to a highly p-doped Si substrate and used as a substrate for the biosensor. Then metal contacts of Ti/Au were subsequently deposited by electron beam evaporation, which was served as the source (S) and drain (D) of the MoS_2_-FET. The specific structure was shown in Figure 3. The PSA antibody (anti-PSA) was directly immobilized on the MoS_2_ surface by the hydrophobicity of MoS_2_. Then, introduction of PSA into the anti-PSA immobilized sensor surface resulted in a current change within the MoS_2_-FET channel. The PSA can be detected by detecting the conduction current between the MoS_2_-FET sensor S and D. And the detection limit of this immunosensor for PSA was 1 pg/ml.

Yoo et al. (2016) designed an epidermal skin-based point of care (POC) device that can monitor PSA in real time. It integrated a MoS_2_-FET biosensor, a readout circuit, and a light-emitting diode (LED) as an indicator into a system. PSA antibodies can be physically adsorbed onto MoS_2_ channels *via* hydrophobic interactions without the need for prior surface chemical treatment. At the same time, when the PSA bound to the antibody, the channel conductivity of the MoS_2_-FET changes accordingly. Therefore, high-sensitivity detection of PSA can be achieved by the change of current in the MoS_2_ channel. And its detection limit (1 pg/ml) was much less than the clinical cut-off.

Similarly, Kaushik et al. (2019) developed a MoS_2_ nanosheets interfaced fiber optic surface plasmon resonance (SPR) sensor for the quantitative analysis of *Escherichia coli* bacteria. The experimental strategy was based on immobilizing the *E. coli* monoclonal antibodies on the MoS_2_ nanosheets *via* hydrophobic interactions. It was demonstrated that the sensing platform can sensitively detect *E. coli* as low as 94 CFU/ml. The target analyte (*E. coli*) has been selectively detected by the developed immunosensor even in the presence of interfering bacteria.

### 2.2 Van Der Waals Force

Van der Waals force is a type of intermolecular force arising from electrostatic interactions between molecules or atoms. Gas molecules, silane coupling agent and single-stranded DNA molecules could be adsorbed on the surface of TMDCs by van der Waals force.

#### 2.2.1 Gas Molecules

The detection principle of gas molecules by TMDCs films can be summarized as the charge transfer mechanism (Zhang et al., 2020; Rahman et al., 2021): according to the different oxidation-reduction characteristics of gas molecules adsorbed on the surface of the films, different charge transfers (including electron donation and electron deprivation) occur on the surface of the TMDCs films. Thereby the conductive properties of the TMDCs films will be changed. TMDCs have a large specific surface area, which allow more atoms to be exposed to the gas; and the semiconducting properties of TMDCs are expected to address the sensitivity, selectivity, and stability issues often encountered in gas sensitive materials (Jeong et al., 2019).

Alagh et al. (2021) reported for the first time on the facile synthesis of 2D layered WS_2_ nanosheets assembled on 1D WS_2_ nanostructures by combining the aerosol assisted chemical vapor deposition (AA-CVD) method with H_2_-free atmospheric pressure CVD. Then the WS_2_ nanosheets was directly integrated into a standard ceramic sensor for an ultrasensitive detection of NO_2_. The detection principle is that when NO_2_ gas molecules are directly adsorbed on the surface of WS_2_, NO_2_ gas molecules will withdraw electrons *via* the valence band, resulting in an overall decrease in the electrical resistance of the film. Subsequently, the measured current will increase under the same voltage and thereby the detection of NO_2_ concentration can be realized. The sensor had reached an unprecedented ultra-low detection limit under 5 ppb. Besides, the response toward NH_3_, H_2_S, H_2_ was studied as a way to assess the potential selectivity of the nanomaterials studied in the detection of NO_2_. And the results showed that the NO_2_ response was significantly higher than the one recorded for any of the other species tested. Additionally, the sensor based WS_2_ nanomaterials had demonstrated its ability to detect 800 ppb of NO_2_ even when operated at room temperature (25°C).

Unfortunately, the gas sensing properties of WS_2_ in its pristine form is weak. However, the heterojunction formation was a promising technique to solve this problem. Kim et al. (2021b) developed a flexible gas sensor that could quickly and sensitively detect CO based on Au-SnO_2_-co-decorated WS_2_-nanosheets. The formation of SnO_2_-WS_2_ heterojunctions could increase the modulation of electrical resistance greatly relative to pristine sensors. At the same time, Au had good catalytic effect towards CO gas, it also could form schottky barrier with WS_2_, leading to enhance response to CO. The flexible gas sensor displayed the highest response and selectivity to CO gas among the different gas sensors investigated under an optimal applied voltage of 4.7 V.

#### 2.2.2 Silane Coupling Agents

Supramolecular interactions, especially the van der Waals force, can drive the formation of physically adsorbed self-assembled monolayers of silane coupling agent onto the surface of TMDCs (Bertolazzi et al., 2018).

Although silane coupling agents cannot react with TMDCs, the deposition of silane on the surface of TMDCs can lead to the physical adsorption of silane. Lee and Park (2017) confirmed that the physical adsorption of silane coupling agents was effective for tunable doping of TMDCs such as WS_2_ and MoS_2_, which could increase charge carrier mobility of TMDCs-FET. Among them, the APTES-MoS_2_ device showed noticeably high on-current and conductance compared to the pristine-MoS_2_ device (Pak et al., 2021). Kang et al. (2015) also showed that the self-assembled monolayer (SAM) doping of TMDCs can improve the performance of its FET. When the MoS_2_-based transistor was doped by APTES, its field-effect mobility was increased from 28.75 to 142.2 cm^2^V^−1^s. In the case of APTES-doped MoS_2_ photodetectors, the photoresponsivity and detectivity were increased 13.8–17.6 times and 12.6–15.2 times, respectively, compared to the undoped MoS_2_ photodetectors. With this doping technology, high-performance TMDCs photodetectors could be developed.

In addition to the above, Zhang et al. (2019b) prepared gold nanoparticle/MoS_2_ composites (AuNPs/MoS_2_) by electrostatic attraction between APTES-functionalized MoS_2_ and citrate-stabilized gold nanoparticles. The AuNPs/MoS_2_ nanocomposites exhibited an excellent performance toward nitrite oxidation. Under the optimal experimental condition, the sensor had a low detection limit of 1.67 µM.

#### 2.2.3 Single-Stranded DNA Molecules

The affinity for DNA probe immobilization was ascribed to van der Waals force (Lan et al., 2019) between nucleobases of single-stranded DNA molecule (ssDNA) and the basal plane of MoS_2_ nanosheets. After probe being hybridized with the complementary DNA (cDNA) to form double-stranded DNA (dsDNA), dsDNA will fall off the basal plane of the MoS_2_ nanosheets due to the different affinity of MoS_2_ for dsDNA and ssDNA (Feng et al., 2018).

**FIGURE 4:**
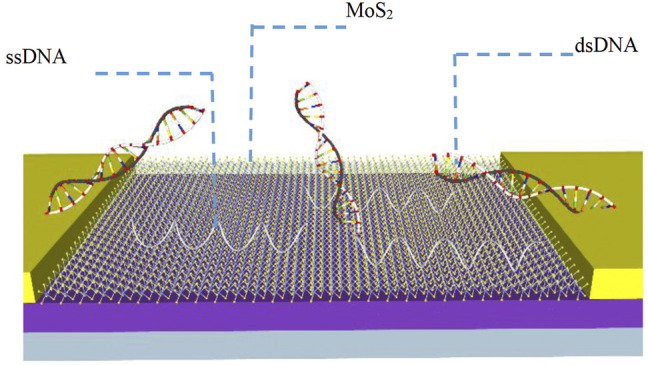
Schematic diagram of MoS_2_ biosensor for detecting DNA (Lee et al., 2015).

Lee et al. (2015) developed a MoS_2_ bio-FET for the sensitive detection of DNA hybridization (Figure 4). The MoS_2_ film served as a sensitive layer for detecting DNA hybridization and an active channel for the solution gate FET structure. Then the DNA probe was directly modified on the surface of MoS_2_-FET by van der Waals force. Due to the van der Waals force between ssDNA nucleobases and MoS_2_, the sensor does not require a gate oxide layer such as HfO_2_, which could significantly improve the coupling between the surface charges of the MoS_2_-FET and the channel conductance. Subsequently, the probe DNA will hybridize with its cDNA to form dsDNA after the target DNA was added, resulting in that the bases were effectively shielded in the dense negatively charged phosphate backbone of dsDNA. The dsDNA will fall off the surface of MoS_2_-FET because of the weak interaction between dsDNA and MoS_2_-FET, causing changes in the threshold voltage and leakage current of MoS_2_-FET. The lower detection limit of this sensor was 10 fM.

TMDCs also have the strong fluorescence quenching ability, which is often used as a sensing platform for DNA fluorescence detection (Wang et al., 2015; Zhang et al., 2015; Kim et al., 2020b; Tao et al., 2021). The ssDNA can be adsorbed on MoS_2_ by the van der Waals force between the nucleobases and basal plane of MoS_2_ nanosheets, the dye molecules labeled on the ssDNA occurred fluorescence quenching attributed to fluorescence resonance energy transfer (Lan et al., 2018). Then the dsDNA formed after cDNA combined with the ssDNA will fall off from MoS_2_ surface resulting in the restoration of the fluorescence. Take advantage of this feature, Zhou et al. (2020) established a sensitive and cost-effective aptameric biosensor based on MoS_2_ nanosheets and aptamer probe for CA15-3 measurement. In this sensing platform, MoS_2_ nanosheets exhibited superior quenching ability to aptamer probe and possessed strong discrimination ability toward aptamer and aptamer-CA15-3 complex, and the lower detection limit reached 3.9 × 10^−3^ U/ml. What’s more, the biosensor may have a promising application prospects in the early diagnosis and evaluation of metastasis as well as recurrence of breast cancer.

### 2.3 Hydrogen Bonding

Hydrogen bonding is a core concept for non-covalent interactions (Wang et al., 2018; Bai et al., 2021). It can be formed between TMDCs and surfactants such as dextran, which plays a crucial role in the aqueous solution exfoliation of TMDCs nanosheets. The common methods for obtaining two-dimensional TMDCs (2D-TMDCs) monolayers films include mechanical exfoliation, chemical vapor deposition (CVD), and liquid exfoliation approach. Of these methods, 2D-TMDCs nanosheets can be scaled up for production under mild conditions by liquid exfoliation while achieving modification (Yim et al., 2018). For example, the addition of surfactants (Li et al., 2022b) in aqueous solution can not only reduce the surface energy of exfoliated 2D-TMDCs nanosheets through intermolecular interactions to achieve effective exfoliation and dispersion of 2D-TMDCs, but also functionalize the surface of 2D-TMDCs nanosheets (Yim et al., 2018). Therefore, this is an approach for the simultaneous exfoliation and functionalization of TMDCs in aqueous solution.

**FIGURE 5:**
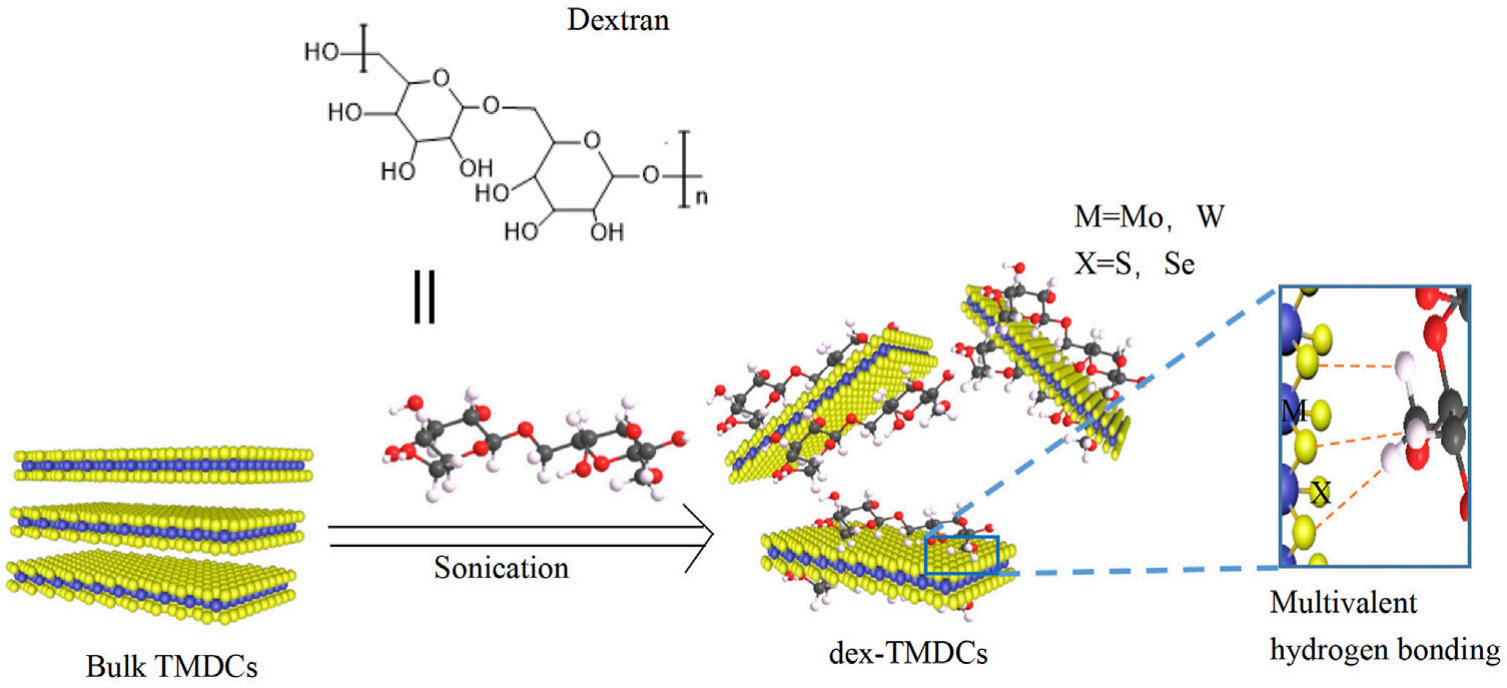
Schematic illustration of the exfoliation and functionalization of TMDCs *via* multivalent hydrogen bonding in an aqueous solution (Kang et al., 2018).

Kang et al. (2018) added dextran to the aqueous solution to achieve simultaneous exfoliation and functionalization of TMDCs nanosheets through multivalent hydrogen bonding generated between the hydroxyl group of dextran and the chalcogens (S or Se) of TMDCs (Figure 5). The resulting dextran/TMDCs hybrids (dex-TMDCs) exhibited a stronger affinity to *E. coli* O157:H7 (*E. coli*) than *E. coli*-specific antibodies and aptamers owing to the recognition capability of a three-dimensional structure of dextran created on the rigid surface of TMDCs nanosheets. The dissociation constant Kd = 11 nM, which was much lower than the reported values of monoclonal antibodies and aptamers.

In addition to dextran, Zong et al. (2017) proved that WS_2_ could be exfoliated efficiently by the assistance of sodium alginate (SA). Upon meeting WS_2_, SA would bind stably to the WS_2_ surfaces *via* strong coordination between carboxyl groups and edge tungsten atoms as well as hydrogen bond between basalplane sulfur and hydroxyl groups. What’s more, the exfoliated WS_2_ had strong mechanical properties superior to natural biocomposites. The discovery of exfoliation ability of low-cost marine polysaccharides may pave a new way to expand the application of TMDCs in biosensors.

Functionalizing the surface of TMDCs by non-covalent interactions such as hydrophobic interactions, van der Waals forces, and hydrogen bonding can ensure the effective coupling of biomolecule charges to the channel, simplify experimental procedures, shorten detection time, and improve the sensitivity of biosensors. However, biosensors based on non-covalent interactions need to face the instability due to weak non-covalent linkages. Compared with non-covalent binding, the covalently constructed sensor can improve the stability.

## 3 Biosensors Based on Covalent Interactions on the Surface of Transition Metal Dichalcogenides

Covalent interaction refers to the link between different substances through chemical covalent bonds. Specifically, the chemical modification of the surface of TMDCs is achieved by generating covalent bonds between other reagents and TMDCs. Covalent bonds are generally stronger than non-covalent bonds (Kumar et al., 2021). Due to the lack of dangling bonds on the TMDC surface, it is difficult to functionalize the surface through covalent interactions. At present, the covalent linkage of TMDCs is mainly achieved through the following three ways: the gold sulfhydryl bond, the π-π coordination covalent bond (Zafar et al., 2022), and sulfur vacancies (Li et al., 2022a) on the surface of TMDCs after pretreatment.

### 3.1 Gold Sulfhydryl Bonds

The metal nanoparticles can be ordered on the surface of TMDCs by direct covalent linkage. For example, AuNPs are assembled on the surface of TMDCs through the gold sulfhydryl bond (Au-S bonds) (Kong et al., 2020) generated between the coordinated structure of MoS_2_ and AuNPs (Parlak et al., 2017). Recent studies have proved that the deposition of metal nanoparticles on TMDCs may change the electronic, optical, and vibrational properties of the TMDCs layer (Abid et al., 2021). Zhang et al. (2016) demonstrated the strain effect induced by metal nanoparticles deposited on the MoS_2_ layer using surface-enhanced Raman scattering (SERS).

The introduction of AuNPs to TMDCs-based biosensors can efficiently accelerate the electron transfer and enhance the detection signal (Huang et al., 2014; Wang et al., 2020b). A novel photoelectrochemical immunosensor had been constructed based on WS_2_ nanosheets and AuNPs for the detection of methylated RNA. The WS_2_ nanosheets with large specific surface area were utilized as photoactive material and AuNPs were immobilized through Au-S bond. The AuNPs were used as the signal amplification unit and immobilization substrate of 4-mercaptophenylboronic acid (MPBA), followed by the specific capture of methylated RNA by anti-m^6^A antibody. Finally, the signal amplification of the sensor was realized by the action of poly(U) polymerase and hexaammonium salt (III) chloride Ru(NH_3_)_6_^3+^ (as a redox probe). The photoactivity of WS_2_ nanosheets was greatly enhanced and the sensitivity was improved by the sensitization of Ru(NH_3_)_6_^3+^. Using visible light excitation and ascorbic acid as the electron donor, the sensitive detection of methylated RNA was achieved by monitoring the photocurrent changes of different concentrations of methylated RNA. Under the optimal experimental conditions, this photoelectrochemical immunosensor showed a good linear relationship with the concentration of methylated RNA in the range of 0.05∼35 nM and the lower limit of detection was 14.5 pM.

### 3.2 π-π Coordination Covalent Bonds

Molecules with pyrene groups, such as 1-pyrenebutanoic acid succinimidyl ester (PASE), could be adsorbed on the surface of MoS_2_ through the π-π coordination covalent bonds between the pyrene group and TMDCs (Huang et al., 2016; Liu et al., 2020b).

**FIGURE 6:**
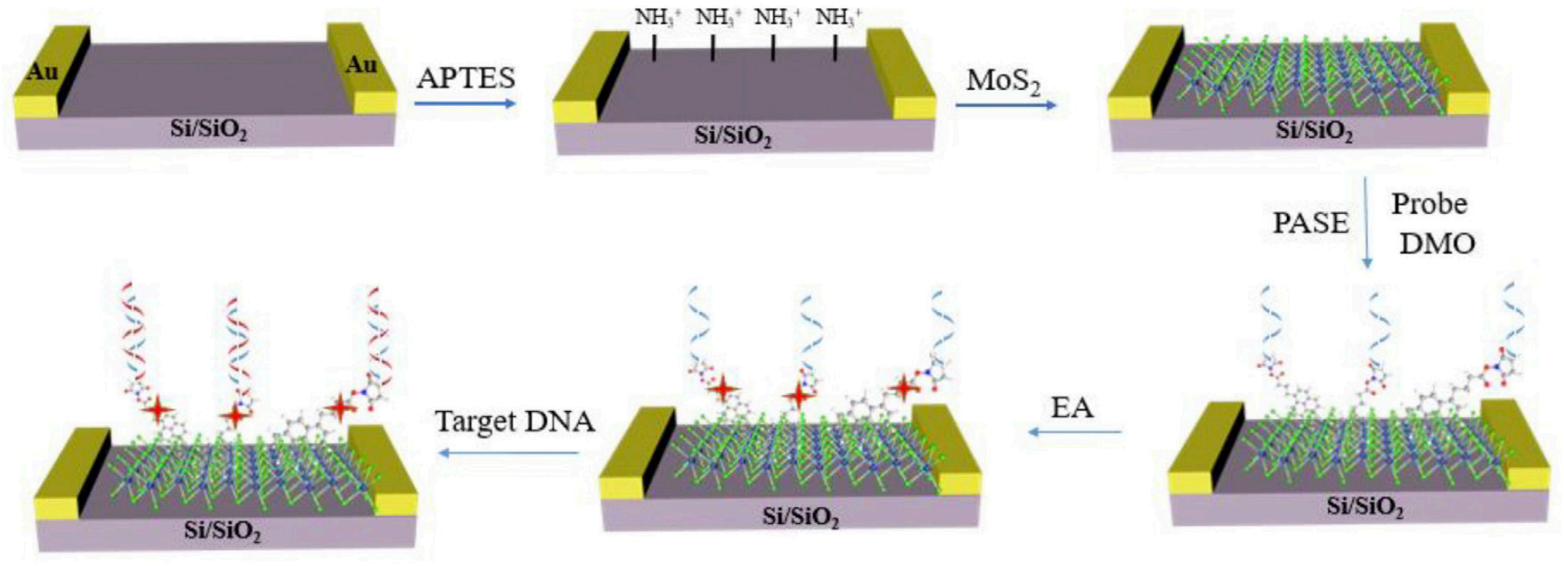
Schematic diagram of MoS_2_ field effect biosensor for DNA detection (Mei et al., 2018).

Mei et al. (2018) fabricated a MoS_2_-FET biosensor chip based on the π-π coordination covalent bonds to detect DNA with high sensitivity and specificity (Figure 6). The PASE was fixed on the MoS_2_ surface though π-π coordination interaction between the MoS_2_ surface and the pyrene group. Then the phosphorodiamidate morpholino oligos (PMO) probe was immobilized on the MoS_2_ surface through the covalently bond between the amino group on PMO and NHS-ester group on the other end of PASE. Compared to DNA probe, PMO enabled a low noise and high sensitivity detection of DNA because it held a neutral backbone of morpholine rings and made a weak impact on their hybridization behavior between PMO and DNA. The detection limit of the sensor can reach 6 fM. And the biosensor showed high sequence specificity capable of distinguishing the complementary DNA from one-base mismatched DNA, three-base mismatched DNA, and noncomplementary DNA. The fabricated MoS_2_-FET biosensor was able to detect DNA in complex sample like serum, making the method potential in disease diagnostics, which demonstrated the advantage of π-π coordination covalent bonds for the biosensor.

The π-π coordination covalent bonds can also be used to develop MoS_2_-FET for detecting low levels of FGF21 in complex biological settings (Gong et al., 2019). MoS_2_ nanosheets obtained by mechanical exfoliation were first transferred to SiO_2_ substrate as the conducting channel of this FET. The PASE was immobilized on the sensor surface using the π-π coordination covalent bonds between the pyrene group at one end of the cross-linker PASE and the MoS_2_. Then, anti-FGF21 was covalently immobilized onto the terminal NHS-ester group of PASE. Subsequently, BSA was introduced to block the unbound sites on PASE. The MoS_2_-FET biosensor demonstrated high sensitivity (10 fg/ml) and specificity, showing its great potential application in disease diagnosis of nonalcoholic fatty liver disease (NAFLD).

Chen et al. (2020) established a MoS_2_/graphene hybrid nanostructure-based biosensor for DNA hybridization detection, combing the advantages of TMDCs and graphene. The MoS_2_/graphene hybrid nanostructure can weaken the Debye shielding effect and avoid the water-induced noiseto improve the sensitivity of the biosensor. The 1-pyrenebutanoic acid succinimidyl ester (PBASE), a molecule with pyrene group, was used as the connector for the probe DNA and hybrid nanostructure. The channel conductance of the MoS_2_-FET would be changed when the target DNA hybrid with probe PMO. Therefore, high sensitive detection of target DNA could be achieved through monitoring the current changes of MoS_2_ channel. The detection limit of this MoS_2_/graphene hybrid nanostructure-based biosensor could reach as low as 10 aM.

### 3.3 Sulfur Vacancies

There are various methods to prepare TMDCs film, such as mechanical exfoliation (Liu, 2021), liquid exfoliation (Raza et al., 2021) and chemical exfoliation (Sun et al., 2017). During the preparation of TMDCs film, the structure of TMDCs usually develop atomic defects due to thermal equilibrium and kinetics of processing (Cavallini and Gentili, 2022), some sulfur atoms are naturally separated from the TMDCs flakes, resulting in sulfur vacancies (Trainer et al., 2022). For example, the crystal structure of MoS_2_ was deformed and internal edges (tears, pinholes, and defects) are clearly visible (Wang et al., 2017; Chen et al., 2022), producing sulfur vacancies using Li^+^ intercalation (Yan et al., 2022; Yang et al., 2022) or ultrasonication. It had been demonstrated that these edge positions (sulfur vacancies) have high molecular affinity for ligand conjugation with thiolates (Kim et al., 2021c; Liang et al., 2021; Osthues et al., 2021).

Based on the sulfur vacancies of MoS_2_, Kim et al. (2014) proposed a highly sensitive, stable, and inexpensive MoS_2_ bulk film chemiresistor sensor for the detection of various volatile organic compounds (VOCs) in the gas phase. The MoS_2_ dispersion was first sonicated to produce sulfur vacancies (defects) in the peripheral edges and internal edges of the MoS_2_ flakes, after which the MoS_2_ dispersion was mixed with a mercaptoundecanoic acid (MUA) solution. Because the MoS_2_ sulfur vacancies had high molecular affinity with the thiol group of the thiol ligand MUA, a carbon sulfur bond (C-S) can be formed to realize the coupling between them. Sensitive membranes were prepared from MoS_2_ and MUA-conjugated MoS_2_ (MUA-MoS_2_) solutions, respectively, and both membranes exhibited high sensitivity (down to 1 ppm) and selectivity for representative volatile organic compounds (VOC) groups (toluene, hexane, ethanol, propionaldehyde, and acetone). It was important to note that the responses of the two were different: the MoS_2_ sensor showed a positive response to oxygen-functionalized VOCs, while the MUA-conjugated MoS_2_ sensor showed a negative response to the same analytes. This study demonstrated that the ligand conjugation successfully increased the functionality of the MoS_2_ matrix. Therefore, this would be a promising approach to construct multifunctional sensor arrays, by coupling multiple thiolated ligands on the MoS_2_ surface.

Similarly, Chiu et al. (2021) treated MoS_2_ solution using ultrasound-assisted liquid phase exfoliation to increase the number of sulfur vacancies on their surfaces. Subsequently, carboxy-MoS_2_ nanocomposites were prepared using the mechanism that the occupation of sulfur vacancies by chlorine atoms led to the formation of covalent bond modifications. The carboxyl-MoS_2_-based biosensor was used successfully to evaluate PAPP-A2 level for fetal Down’s syndrome screening in maternal serum samples and the detection limit was 0.05 pg/ml.

In general, functionalization of TMDCs surface by covalent bonds is more stable than non-covalent bonds, resulting in stronger binding to biorecognition molecules. Therefore, the biosensors constructed based on covalently modified TMDCs have better performance in terms of stability and repeatability. The stable modification of the biometric element also improves the sensitivity of the sensor. However, compared with non-covalent modification methods, covalent modification-based sensors increase the complexity of experimental operation and detection time.

## 4 Summary and Prospects

During the fabrication of biosensors, the immobilization of sensitive elements is a critical step in determining the sensor performance. For TMDCs-based biosensors, the surface functionalization of TMDCs is a significant step related to the performance of the recognition element. This paper reviewed the different modification methods of TMDCs for the TMDCs-based biosensors fabrication and the biosensors’ research progress.

The surface functionalization of TMDCs mainly includes non-covalent and covalent interactions. Non-covalent modification methods include hydrophobic interactions, van der Waals forces and hydrogen bonding. Covalent modification methods include gold sulfhydryl bonds, π-π coordination covalent bonds, and sulfur vacancies on the surface of TMDCs after pretreatment. Non-covalent interaction could avoid the additional complexity involved in surface chemical treatment, and simplifying the biosensor fabrication and shortening the detection time. Compared with non-covalent bonds, covalent bonds are more stable for the functionalization of the surface of TMDCs, resulting in stronger binding to biorecognition molecules. Biosensors based on covalently modified TMDCs have better performance in terms of stability and reproducibility. The stable modification of the biometric element also improves the sensitivity of the sensor. However, compared with non-covalent modification methods, covalent modification-based sensors increase the complexity of experimental operation and detection time.

In summary, non-covalent and covalent interactions have their own advantages and disadvantages in surface functionalization methods for TMDCs-based biosensors. How to combine their advantages and greatly improve the sensitivity and stability of biosensors will be an important research direction for the development of biosensors based on TMDCs in the future. TMDCs are an excellent material for fabricating biosensors due to their specific physical and chemical properties. The diversity of modification methods can undoubtedly promote the wide application of TMDCs in biosensors, but overcoming their inherent defects is still a challenge for scientists.

Firstly, the low conductivity of pristine TMDCs in biosensors remains a problem that cannot be ignored. The most current solution is to incorporate other nanomaterials (i.e., graphene, AuNPs, BP, etc.) onto TMDCs to enhance the sensing performance. For example, the heterojunction formed by the combination of TMDCs and graphite can simultaneously solve the problem of zero band gap by graphite and low electrical conductivity of TMDCs.

Studies have demonstrated that a single layer of TMDCs can provide a higher on/off ratio when applied to field effect transistor configurations. But the preparation methods of high-quality monolayer TMDCs film needs to be further studied, and the performance of monolayer film is easily disturbed by the external environment, which seriously hinders its application in biosensors. Therefore, it is important to synthesize high-quality TMDCs monolayer films and functionalize their surfaces using the suitable modification method in order to achieve high-sensitivity and reliable biosensor construction.

On the other hand, due to their unique nanostructures, large specific surface area coupled with their unique semiconductor properties with tunable band gaps, TMDCs can also be used as an outstanding nanoenzyme material. The emergence of TMDCs nanozymes provides an opportunity for targeted drug delivery for precision cancer therapy. Different anticancer effects can be achieved by self-assembly of diverse substances on the surface of TMDCs nanozymes *via* non-covalent and covalent interactions. What’s more, the broad compatibility with various substrates, strong mechanical strength, and excellent elasticity for mechanical deformation make TMDCs one of the popular nanomaterials for wearable biosensors. It can be expected that the performance of the TMDCs-based sensor will be further improved in future research. And the multifunctional TMDCs-based biosensors will have broader application prospects with the rapid development of TMDCs nanomaterials.
